# Risk factors and management of hyperuricemia after renal transplantation

**DOI:** 10.3389/fsurg.2022.956213

**Published:** 2023-01-06

**Authors:** Xiaoyu Zi, Xi Zhang, Chuan Hao, Zhenxing Wang

**Affiliations:** ^1^ Department of Rheumatology, The Second Hospital of Shanxi Medical University, Taiyuan, China; ^2^ Department of Urology, The Second Hospital of Shanxi Medical University, Taiyuan, China

**Keywords:** risk factors, uric acid, hyperuricemia, renal transplantation, drug induced

## Abstract

Hyperuricemia (HUA) is a common complication after renal transplantation. Currently, there is no uniform consensus on factors which increase the risk for and treatment of HUA in renal transplant recipients. The purpose of this review is to summarize current and proposed risk factors and strategies to manage HUA after renal transplantation in order to assist renal function protection and prolong graft survival time.

## Introduction

Hyperuricemia (HUA) is a common complication after renal transplantation (1), and the upper limit of the HUA incidence in renal transplant recipients reported over 80% with the wide applications of cyclosporine (1). HUA is defined as a serum uric acid (UA) level greater than 7.0 mg/dl in men and 6.0 mg/dl in women (2). UA reportedly causes oxidative damage in various tissues (3). HUA induces kidney damage traditionally thought to occur from inflammation brought on by sodium urate crystals deposition in renal tissue (4, 5). There are a number of risk factors associated with post transplant HUA including, older age, male gender, calcineurin inhibitors, diuretics, hypercalcemia, lower estimated glomerular filtration rate (eGFR), long-term pre transplantation dialysis and the presence of pre transplant hyperuricemia (1, 6–9). Elevated serum UA levels can decline long-term eGFR and worsen kidney graft function (10). More specifically, HUA was found to be associated with an increase in graft loss, short term graft survival and a higher risk of cardiovascular disease and mortality (11), resulting in poor quality of life and a dramatic increase in the economic burden of renal transplant recipients.

This review aims to deliver a comprehensive and accurate understanding of the risk factors for HUA after renal transplantation, as well as provide new insights into individualized prevention strategies and therapy protocols for HUA in renal transplant recipients.

## Production and excretion of uric acid

### Production of uric acid

UA is an end product of the digestion of exogenous purines derived largely from animal proteins in the liver, intestines, and vascular wall (12). In addition, UA is also the byproduct of the degradation of endogenous purines of damaged, dying, and dead cells that have nucleic acids, adenine, and guanine (12, 13). Adenine and guanine are converted to inosine and guanosine through deamination and dephosphorylation, respectively. The enzyme purine nucleoside phosphorylase subsequently converts inosine and guanosine to hypoxanthine and guanine, which are both converted to xanthine by xanthine oxidase (XO) through oxidation of hypoxanthine and deamination of guanine by guanine deaminase (14). XO further oxidizes xanthine to UA (15).

### Excretion of uric acid

The kidney excretes approximately 70% of UA produced daily, and the remaining 30% is excreted *via* the intestine by bacteria cleaving UA into waste substances that are ultimately eliminated in feces (12, 16). Renal urate excretion mainly involves three processes: filtration, reabsorption and secretion (17). As reported, the proximal tubule is the main site of UA reabsorption and secretion, and approximately 90% of UA is reabsorbed into blood, which is primarily accomplished at the proximal tubular level by transporters that exchange intracellular anions for UA (18). Various transporters play a significant role in renal reabsorption of UA (13, 19). The urate transporter 1 (URAT1) protein is the product of the SLC22A12 gene, which is mainly located on the apical (luminal) side of the proximal tubule; while glucose transporter 9 (GLUT9) encoded by SLC2A9, which has roles similar to those of URAT1, is present on the basolateral side of proximal tubule cells (13, 15, 19). These two transporters are the main targets of present uricosuric drugs (19). Apart from these, organic anion transporter 4 (OAT4) and OAT10, respectively encoded by the SLC22A11 and SLC22A13 genes, are both expressed on the apical membrane of the proximal tubule and have similar roles as URAT1 (13).

The kidney also expresses secretory transporters to excrete UA. OAT1, which is encoded by the SLC22A6 gene and OAT3, encoded by SLC22A8, are both present on the basolateral membrane of renal proximal tubules, and they are principally involved in luminal secretion of UA (14), transporting urate from the interstitial fluid into proximal tubule cells (13). Since the balance of production and elimination of UA determines the level of UA in the body, an increased UA production and/or impaired renal UA excretion, causes the development of HUA (12).

## Risk factors for HUA after renal transplantation

The risk factors for HUA after renal transplantation can be broadly classified into demographic characteristics, metabolism-related factors, drug use and other factors.

### Demographic characteristics

Factors such as age, sex seem to affect UA in kidney transplant recipients. A study reported that reduced renal function appears likely to be responsible for the increase in UA in aged people (20). Kevin et al. conducted a retrospective cohort study of 59,077 renal transplant patients, and found that an older recipient age significantly contributed to increased risks of new-onset gout after renal transplantation (21). A retrospective cohort study of 302 renal transplant recipients showed that hyperuricemic patients were predominately older age (22). Therefore, older age is a risk factor for HUA after renal transplantation, but the detailed mechanisms remain unknown.

Many studies have shown that the development of HUA after renal transplantation is associated with the male sex (8, 22). Malheiro et al. found that hyperuricemic patients were overwhelmingly male of 302 renal transplant patients (22). This gender bias could be explained by the fact that estrogenic compounds enhance renal urate excretion in women, possibly reducing the active renal urate transporters resulting in less tubular UA reabsorption (7, 23).

### Metabolism-related factors

HUA is associated with metabolism-related factors, such as obesity, diabetes mellitus, hypertriglyceridaemia and hypertension. Metabolic risk factors could negatively affect the graft function and cause graft loss (24). Previous studies showed that an increase in Body Mass Index was directly related to a higher risk for HUA, and obesity could contribute to elevated UA levels by decreasing urinary UA excretion (22, 25, 26). A study of 302 renal transplant patients showed that increasing Body Mass Index is a risk factor for HUA after renal transplantation (22). The most probable reason for this link is that obesity is characterized by insulin resistance, which activates the sympathetic nervous system and renin–angiotensin system and then produces lactic acid, which competitively inhibits UA secretion and ultimately causes higher serum UA (26).

As extensively reported, diabetic nephropathy causes renal structural changes (including glomerular hypertrophy, glomerular basement membrane thickening, partial glomerulosclerosis and extensive glomerulosclerosis) and dysfunction of renal tubular excretion (26, 27). HUA in renal transplant recipients with diabetes is possibly caused by the reduced eGFR and the increased reabsorption of renal tubules (26, 27). So diabetes mellitus may increase the risk of HUA after renal transplantation.” after the references number (26, 27).

The activity of glyceraldehyde 3 phosphate dehydrogenase which is reduced by hyperglycemia and hyperlipidemia can also enhance UA synthesis (26). At present, there is not much evidence available to fully elucidate whether hypertriglyceridaemia is a risk factor for HUA after kidney transplantation. Some studies have shown that UA progressively increases with increasing serum triglyceride levels (6, 28). The exact mechanisms, however, remain unknown. It is generally known that adenosine-triphosphate is needed for fatty acid synthesis and triglyceride anabolism, and depletion of adenosine-triphosphate can lead to the accumulation of adenosine monophosphate and overproduction of UA (28). Thus, hypertriglyceridaemia is a probable risk factor for HUA after kidney transplantation.

Many studies have reported that hypertension is associated with HUA in renal transplant recipients (8, 25, 29). Hypertension-caused renal ischemia could enhance reabsorption of UA by the renal proximal tubule (30). Experimental studies have reported that HUA induces hypertension and kidney injury *via* renal vasoconstriction mainly induced by endothelial dysfunction and the activation of the renin-angiotensin system (29). However, further studies are still needed to confirm a bidirectional link between HUA and hypertension.

### Drug use

Currently, calcineurin inhibitors (CNIs) are the standard immunosuppressive therapy after renal transplantation (31). Previous studies have observed that CNIs, including tacrolimus and cyclosporin A (CsA), are risk factors for HUA after renal transplantation (1, 22, 32). Calcineurin is a significant target of immunosuppressive therapy with the main aim of inhibiting T-cell proliferative responses to donor alloantigens (33). CsA and tacrolimus are widely used in clinical transplantation (33), and their primary mechanism of pharmacological function mainly includes inhibiting phosphorylation of nuclear factor of activated T- cell which consequently reduces T-cell activation and proliferation mediated by Interleukin-2 and inhibits T cell-mediated rejection (34, 35). CNIs are nephrotoxic agents, and HUA is a common complication of CNI therapy (36, 37). Cyclosporine-induced HUA has been associated with the reduction of urinary clearance of UA due to increased proximal tubular reabsorption, decreased tubular secretion, and decreased GFR (22, 38, 39). Tacrolimus has also been found to be associated with HUA (38, 40), but at a less frequency compared to CsA (32). For tacrolimus, the effect on UA levels is not as well established and is only known to decrease the excretion and glomerular filtration of UA caused by vasoconstriction (41, 42).

Apart from that, renal transplant recipients are prone to hypertension and edema, so diuretics and other antihypertensive drugs are commonly used in their management (38). A large case-control study of 74,768 patients from the United Kingdom reported that beta blockers and diuretics were related to a higher risk of HUA (43). Since, there is not much evidence to prove that the use of beta blockers is a risk factor for HUA after renal transplantation, a detailed mechanism needs further investigation. Multiple studies have reported that diuretics were directly related to a higher risk for HUA after renal transplantation (7, 8, 32). Among these, thiazide diuretics and loop diuretics can interact with renal OAT; they enter the proximal tubular cells from the blood by OAT1 and OAT3, which could probably compete with UA, causing the reduced secretion of UA (30, 44, 45). Moreover, diuretics also decrease UA excretion, possibly by causing blood volume depletion with a consequent increase in proximal tubular reabsorption of UA (38).

### Hypercalcemia

A retrospective study revealed that of 356 renal transplant recipients, 55 (15.45%) had HUA and their serum calcium concentrations were significantly associated with increased UA levels (46). Therefore, hypercalcemia highly increases the risk of HUA after kidney transplantation. It was reported that hypercalcemia can induce kidney injury, and the pathophysiologic mechanisms may be vasoconstrictive processes, intra-tubular calcifications, interstitial nephritis and hypovolemia (47). Additional studies are needed to explore the effects of serum calcium concentrations in HUA after renal transplant recipients.

### Lower eGFR

Although, the incidence of rejection has already decreased with the development and administration of immunosuppressants, chronic cellular or humoral rejection unavoidably occurs (48). Renal ischemia reperfusion injury is also a common and unavoidable event after renal transplantation (49). The factors mentioned could cause renal graft injury, which probably causes reduced eGFR. A study showed significantly increased odds of HUA linked to a decline in eGFR values in renal allograft recipients (22). Numakura et al. found that decreased eGFR (<60.0 mL/min /1.73 m^2^) was a risk factor for HUA at 1 year after renal transplantation (8). Since UA is excreted mainly by the kidney, a rise in serum UA occurs as the GFR falls (7, 8). Conversely, HUA induces arteriolopathy of preglomerular vessels and impairs the autoregulatory response of afferent arterioles, which causes glomerular hypertension and reduced GFR (8), ultimately leading to loss of graft function. Thus, HUA is both a result of and a cause of reduction of eGFR in renal transplant recipients.

### Long-term pre transplantation dialysis

Maintenance hemodialysis is the commonly used kidney replacement methods in end-stage kidney disease (50). Studies have shown that short-term hemodialysis significantly reduces UA levels by approximately 60% without additional ULT in patients with end-stage kidney disease (50, 51). However, some studies have shown that long-term pre transplantation dialysis (>36 months) is associated with HUA after renal transplantation (8, 32). One reliable interpretation is that hyperparathyroidism, a common complication in dialysis patients, causes HUA by increased urate absorption (52). Another possible explanation is that hypoxia and oxidative stress contribute to an increase in hypoxanthine, which can be converted to UA by xanthineoxidase. Hemodialysis patients are undoubtedly exposed to potential hypoxia and oxidative stress during the hemodialysis process (53). Thus, long-term pre transplantation dialysis is a risk factor for HUA after kidney transplantation.

### The presence of pre transplant HUA

As we have already mentioned, although short-term hemodialysis significantly reduces UA levels, long-term pre transplantation dialysis may cause HUA. Previous studies have demonstrated that a preexisting history of HUA is related to HUA after kidney transplantation (1, 32, 46). The mechanisms of HUA-induced inflammation, oxidative stress, endothelial dysfunction, and renal fibrosis (54) may be associated with HUA post transplantation.

As mentioned above, numerous risk factors act separately or synergistically to induce HUA after renal transplantation. Serum UA concentration elevated to pathological levels may lead to renal damage (55), which may affect renal graft function. Thus, HUA may contribute to the reduced renal allograft function and eventually cause graft loss. In the following section, we will discuss the progress in HUA management after renal transplantation.

## Management of HUA after renal transplantation

### At-risk populations

Keeping in view all the factors discussed so far, it can be concluded that recipients are at a high risk of HUA after renal transplantation. Improving treatment adherence for metabolism-related factors, selecting rational CNIs and standardizing the utilization of drugs that inhibit UA excretion are particularly important for reducing the risk of HUA in recipients. When the condition allows, drugs that increase UA levels should be discontinued, such as loop and thiazide diuretics, beta-blockers (56). Moreover, HUA can be caused using CNIs, especially CsA (57). Thus, individualized immunosuppressive protocols that focus on cellular rejection as well as humoral rejection during renal transplantation promise a better balance between necessary control of alloreactivity (58) and reduced incidence of HUA. Additionally, new therapeutic strategies targeting renal ischemia reperfusion injury to extend graft survival include machine perfusion, exogenous administration of mesenchymal stem cells, and ex vivo preservation using preservation solutions saturated with alternative gases (49). This may reduce renal injury during renal transplantation and maintain normal UA excretion.

### Lifestyle intervention

HUA may be caused by UA overproduction as a result of a high purine diet, fructose ingestion, alcohol consumption, and genetic causes such as hypoxanthine-guanine phosphor-ribosyl-transferase deficiency and phosphor-ribosyl-pyrophosphate synthetase hyperactivity (59). A critical review reported that the metabolism of fructose can cause elevated UA levels due to decreased UA excretion and increased hepatic adenosine-triphosphate degradation to adenosine monophosphate, a UA precursor (16, 59). A 2018 meta-analysis showed that several kinds of food: soft drinks, wine, liquor, beer and meat (lamb, pork, beef) contributed to raised UA levels (60). Thus, lifestyle intervention may play a pivotal role in the prevention of HUA after renal transplantation. Lifestyle interventions including exercise, weight reduction, low consumption of purine-rich meat, limiting the intake of alcoholic beverages, and avoiding high fructose intake (including sweetened soft drinks and energy drinks), are recommended for all HUA patients (61). Remarkably, gradual weight loss is more beneficial than a drastic reduction, as abrupt weight loss contributes to ketosis, which increases UA reabsorption *via* URAT1, resulting in increased serum UA (62). In addition, profuse sweating exercise causes a reduction in urinary UA excretion and results in increased serum UA after exercise; therefore, drinking enough fluids to prevent dehydration and maintain sufficient urine output is recommended (63). Moreover, alkalization of urine *via* manipulation of food materials can promote the removal of UA (64). Dairy products, such as vegetables, fruits (less sugary ones), legumes, nuts and whole grains are beneficial for the comorbidities of HUA and may also help prevent HUA by reducing insulin resistance (65).

### Treatment for HUA after renal transplantation

It has been reported that lowering UA can prevent renal functional loss and vascular injury (66). UA-lowering treatments (ULTs) can be classified as direct-acting and indirect-acting agents. For direct-acting agents, there are two major classes of ULT agents widely used in clinical practice: one suppressing UA synthesis and another promoting UA excretion (67). Most guidelines do not recommend treating asymptomatic HUA, but drug therapy is officially allowed in asymptomatic HUA according to the Japanese guidelines on management of HUA, particularly when serum UA level reaches 8.0 mg/dL or more in cases with comorbidities such as hypertension, renal impairment (68).

### Uricostatic drugs

Allopurinol is an XO inhibitor and its major metabolite, oxypurinol, is predominantly eliminated by the kidney; thus it is required to adjust the dose in renal impairment. Patients with renal impairment may also have a higher risk of life-threatening allopurinol hypersensitivity syndrome (69). For patients with creatinine clearances above 60 ml/min, the allopurinol should decrease serum UA to below 6.0 mg/dl in individualized dosage (70). It should be noted that administering allopurinol in renal transplant recipients receiving azathioprine has the danger of fatal pancytopenia. Azathioprine is a commonly used immunosuppressive agent for the prevention of graft rejection in renal transplant patients (71). Azathioprine is a prodrug, and its active form is 6-mercaptopurine. 6-mercaptopurine has three metabolic pathways in the body: by thiopurine methyltransferase into 6-methylmercaptopurine, by XO into 6-thiouracil, and by hypoxanthine guanine phosphoribosyltransferase into 6-thioguanine (72). Severe anemia is a recognized but uncommon manifestation of azathioprinerelated myelosuppression (73). It was reported that an interaction between azathioprine and allopurinol inhibiting the XO pathway of azathioprine metabolism, was the main reason for severe anemia (74). Therefore, when using allopurinol with azathioprine, a lower dose is suggested, with weekly complete blood counts in the first month to monitor for toxic adverse effects, simultaneously (72, 75).

Febuxostat, which is a novel nonpurine-selective XO inhibitor, is metabolized primarily by glucuronide formation and oxidation in the liver (76), and strongly inhibits both the oxidized and reduced forms of XO at low concentrations; therefore, there is no need to adjust its dose in mild to moderate renal impairment (77). Although febuxostat should be administered with caution in patients with severe renal dysfunction (GFR < 30 ml/min), its metabolic characteristics are more advantageous than allopurinol (78).

### Uricosuric drugs

Benzbromarone, a URAT-1 inhibitor, generally shows high efficacy and safety even for patients with chronic kidney diseases (79). The drug was not licensed in the United States and numerous European Union nations due to its severe idiosyncratic hepatotoxic side effects (80); however, in some HUA patients with impaired renal function, benzbromarone is significantly effective in lowering UA levels (81). However, liver toxicity due to benzbromarone is still a concern (82).

Probenecid, inhibiting OAT and URAT1, exhibits less potent hypouricaemic effects than benzbromarone (80). Notably, probenecid cannot be used in patients with urolithiasis and a GFR < 50 ml/min when adverse events and drug interactions frequently occur (83). Thus, probenecid is not recommended for patients with severe renal impairment (eGFR < 30 ml/min/1.73 m^2^) (82).

Arhalofenate, a novel anti-inflammatory uricosuric agent, mainly decreases UA by inhibiting URAT1, OAT4 and OAT10, and reduces the release of interleukin-1β stimulated by monosodium urate crystals through the peroxisome proliferator-activated receptor gamma pathway (84, 85). The dual mode of action of arhalofenate exhibits a substantial advantage over other ULTs (86). Arhalofenate was reported to be more potent than probenecid in uricosuric activity (85), and could be a potentially attractive novel agent for HUA therapy for renal transplant recipients.

Tranilast, also an anti-inflammatory drug, exerts uricosuric properties by interacting with URAT1, GLUT9, OAT4, and OAT1 (87). Preclinical trials of tranilast in healthy volunteers showed that not only did it have a urate-lowering effect but also reduced urate crystal-associated inflammation (88), making it an ideal therapeutic agent for HUA after renal transplantation. Nevertheless, the adverse effects of tranilast, such as liver injury, eosinophilic cystitis, eosinophilic polymyositis and immune thrombocytopenia, have been reported (83). Therefore, a better knowledge of the kidney tolerance of these new uricosuric drugs is urgently needed to determine their risk: benefit ratio (82).

### Indirect UA-lowering treatment

HUA is usually accompanied by various comorbidities, including cardiovascular disease, metabolic syndrome and other conditions (89, 90). When making treatment regimens for these comorbidities, drugs that increase renal UA excretion are recommended, such as calcium channel inhibitors or losartan for hypertension, glitazones and biguanides for diabetes (sodium-glucose cotransporter 2 inhibitors, SGLT-2), and fenofibrate or atorvastatin for dyslipidemia (56, 90–92).

Losartan has a hypouricaemic effect among antihypertensive medications (93). Hyon et al. showed that losartan and calcium channel blockers may be protective against the risk of HUA among people with hypertension due to their uricosuric properties (92, 94).

SGLT-2 inhibitors are a new class of antidiabetic drugs that increases urinary glucose excretion by reducing renal glucose reabsorption in the proximal convoluted tubule (95). Several clinical trials of patients with and without type 2 diabetes have shown that SGLT-2 inhibitors have consistently favorable cardiovascular and kidney effects (96). The SGLT-2 inhibitors (dapagliflozin, empagliflozin, canagliflozin, etc.) reportedly had a UA-lowering effect by increasing the glucose concentration in renal tubules and excreting UA at the S1 segment of the proximal tubule, both of which enhanced the excretion of UA (66, 97). Moreover, SGLT-2 inhibitors have added benefits, such as blood pressure control, weight loss, and possible lipid lowering effects, to meet uncertain clinical needs (98). However, SGLT-2 inhibitors have not been approved for renal transplant recipients, and may have great application prospects in renal transplant recipients with type 2 diabetes and HUA to reduce the risk of cardiovascular death (67).

Fenofibrate, a peroxisome proliferator-activated receptor alpha agonist, has lipid-modifying effects on high triglyceride and reduces the microvascular complications of diabetes (99). Fenofibrate was recommended as part of a comprehensive strategy to lower UA concentrations, as it decreases UA by promoting UA clearance (91). Thus, the renal transplant recipients with HUA with different comorbidities should use corresponding indirect UA-lowering drugs to enhance the effect of UA lowering therapy.

## Conclusions

Renal transplant recipients are particularly vulnerable to HUA since there are several risk factors that contribute to deteriorating renal function. HUA severely impairs renal function and ultimately results in graft loss. Thus, the management of risk factors for HUA and lifestyle interventions in renal transplant recipients are critical to prevent renal damage caused by HUA and are extremely important for prolonging the survival time of grafts. Future studies need to focus on the mechanisms of HUA-induced renal injury in renal transplant recipients, which will further guide effective treatments for HUA after renal transplantation.
